# Treatment of Liquid Digestate by Green Algal Isolates from Artificial Eutrophic Pond

**DOI:** 10.3390/molecules27206856

**Published:** 2022-10-13

**Authors:** Ewelina Sobolewska, Sebastian Borowski, Paulina Nowicka-Krawczyk, Katarzyna Banach

**Affiliations:** 1 Department of Environmental Biotechnology, Faculty of Biotechnology and Food Sciences, Lodz University of Technology, Wólczańska 171/173, 90-530 Lodz, Poland; 2 Department of Algology and Mycology, Faculty of Biology and Environmental Protection, University of Lodz, Banacha 12/16, 90-237 Lodz, Poland

**Keywords:** liquid digestate, nutrient removal, *Microglena* sp., *Tetradesmus obliquus*, *Desmodesmus subspicatus*

## Abstract

The ability of aquatic microalgae to treat the liquid digestate obtained from the anaerobic digestion of plant waste was investigated. Microalgae were isolated from natural environment for a laboratory-scale cultivation and were then used to remove nutrients and organic contaminants from the liquid digestate. It was shown that the microalgae consortia (*Tetradesmus obliquus*, *Microglena* sp., *Desmodesmus subspicatus*) could reduce nitrogen, phosphates, and total COD by up to 70%, 57%, and 95%, respectively. A new algae genus *Microglena* was isolated, which in a consortium with *Tetradesmus obliquus* and *Desmodesmus subspicatus* exhibited a high efficiency in the removal of both organic contaminants and nutrients from the liquid fraction of digestate.

## 1. Introduction

Anaerobic digestion (AD) is an important technology in the context of a circular economy that allows for the processing of organic waste into biogas and nutrient-rich digestate. Biogas is the crucial product of anaerobic digestion, which can either be used to generate heat and electricity or be converted into biomethane via the membrane upgrading process. However, special attention should be paid to the digestate which, when properly processed, can be reused in agriculture or industry. Unprocessed digestate creates a great ecological hazard due to the high concentrations of organic substances, nutrients, or even potential toxic elements. Inadequate digestate treatment or its improper application to the soil may contribute to eutrophication processes, soil pollution, groundwater contamination, and unfavorable greenhouse gas emissions. Therefore, the treatment and recirculation of digestate at biogas plants considerably reduces the aforementioned problems and is in line with the closed-loop concept [1,2,3].

The anaerobic digestate can be separated into a liquid fraction (80–90% of the mass) that is rich in nitrogen and phosphorus and a solid fraction (10–20% of the mass) that contains large amounts of fibers. The separation of these two fractions from each other allows for their independent treatment. Compared to liquid digestate, solid digestate contains less water and is more stable, allowing for easier transportation and storage. The solid digestate can be further processed to create value-added products such as organic fertilizers, compost, nanocellulose, pyrochar, or heat. In turn, the liquid digestate can be returned to the biogas production process and be reused for the dilution of raw materials. This can reduce water consumption and the amounts of wastewater streams generated in a biogas plant. However, to enable its reuse for waste processing and biogas production, the liquid digestate must be properly treated to remove most of organic pollutants and nutrients in order to meet the EU requirements [1,3,4].

The treatment of liquid digestate is a multifaceted problem resulting from the high content of carbon-, nitrogen-, and phosphorus-containing compounds with a very low C/N ratio, which are typically difficult to remove using traditional methods. The simplest method of digestate utilization is its direct application to soil. However, this may lead to water eutrophication as well as a chemical and biological contamination of the soil, thus reducing its productivity in the long run. Additionally, ammonia volatilization and odor release deteriorate the air quality in the vicinity of the biogas installations. The growing number of biogas plants is often accompanied with huge volumes of the digestate being stored in lagoons or applied to soil in excessive doses. Consequently, a drop in acceptance for these installations is being observed, which, in turn, forces the need to look for new and effective methods of digestate processing [4,5].

Microalgae have enormous potential in the production of biofuels (biodiesel, biohydrogen, and biogas) and bioproducts (lipids, carbohydrates, and pigments), and they can be applied for the treatment of digestate [6,7]. These organisms are characterized by a relatively high growth rate, the ability to live under difficult environmental conditions, resistance to pollution, high efficiency of the photosynthesis process, and the ability to bind CO_2_. However, the cultivation of microalgae is a an expensive process due to their high nutrient requirements. In this context, the liquid digestate, which is rich in nitrogen, phosphorus, and organic carbon, can be a good environment for the growth of microalgae. In fact, many types of microalgae show high tolerance to the wastewater environment and show the ability to absorb nutrients from the liquid fraction of the digestate, thus leading to its purification. Microalgae can utilize dissolved carbon dioxide and nutrients in the form of ammonia, nitrates, nitrites, and phosphates, which makes these microorganisms suitable for the treatment of the liquid digestate. Furthermore, microalgae are also known for their ability to absorb and convert organic carbon into cellular components, such as sugars or fats. It is also believed that microalgae can remove heavy metals and other harmful compounds. Research on the cultivation of microalgae in wastewater or waste streams and their participation in the reduction of harmful compounds is of interest to many scientists. The most commonly used genera for this application have been *Scenedesmus*, *Chlorella*, *Chlamydomonas*, and *Desmodesmus*. Previous reports have shown that these microalgae may contribute to a decrease in carbon, nitrogen, and phosphorus contents in wastewater [8,9,10,11,12].

However, only some studies have described the application of algae for the treatment of liquid digestate derived from biogas production processes. Xu et al. [13] reported a high efficiency of *Tetradesmus obliquus* (formerly known as *Scenedesmus obliquus*) in removing COD (chemical oxygen demand), phosphorus, and total nitrogen from piggery anaerobic digestate. Kisielewska et al. [14] proved that *Chlorella vulgaris* is capable of reducing total nitrogen and phosphorus from liquid digestate obtained from maize silage and stillage. The level of reduction was dependent on the digestate treatment method (distillation or centrifugation) and was higher in the distilled digestate. Massa et al. [15] demonstrated the high removal rate of pollutants by *Botryococcus braunii* and *Tetradesmus obliquus* from liquid digestates of animal, plant, and municipal origin. *Scenedesmus acuminatus* (now *Tetradesmus lagerheimii*) was also successfully applied for the treatment of the digestate derived from the anaerobic digestion of pulp and paper industry biosludge. The investigated microalgae partially removed soluble COD and color, whereas sulfates, phosphates, and ammonia were almost completely removed [16].

The aim of the current research was to examine the ability of microalgae isolated from the natural environment to treat liquid digestate derived from the anaerobic digestion of vegetable waste. The research consisted of two main stages: (1) the isolation and cultivation of microalgae on a laboratory scale; and (2) the use of isolated microalgae for the treatment of liquid digestate. A novel aspect of the research is the successful isolation of the *Microglena* algae, which in a consortium with *Tetradesmus obliquus* and *Desmodesmus subspicatus* demonstrated the ability to effectively remove organic contaminants and nutrients from the liquid digestate. *Microglena* sp. has not yet been documented in studies concerning liquid digestate treatment methods. These preliminary studies have also proven that successful liquid digestate treatment systems do not require the utilization of commercial microalgal culture collection strains and can be designed using environmental isolates.

## 2. Results and Discussion

### 2.1. Selection of the Liquid Fraction of Digestate Used for the Tests

The characteristics of the tested digestates are summarized in Table 1. In the nomenclature used, GS refers to the liquid digestate collected from the municipal wastewater treatment plant in Lodz, Poland, whereas MK1 and MK2 are liquid digestates from the pilot installation at Warmia Fruit and Vegetable Processing Company Ltd. in Kwidzyn, Poland, as explained in Section 3.1. The MK2 digestate exhibited the greatest concentration of suspended solids as well the highest values of color and turbidity. In contrast, the GS effluent was mainly polluted with soluble contaminants with a very low pH value of 4.43 as a result of using coagulants and polyelectrolytes during the dewatering process at the wastewater treatment plant. The total and soluble COD as well as the concentration of volatile fatty acids were at comparable levels in all the digestates. Regarding nutrient content, the MK2 digestate had the highest amounts of ammonium nitrogen (580.00 mg/L) and nitrates (1016.67 mg/L). In contrast, the amount of phosphates (810.00 mg/L) was the highest in the GS liquid as a result of the chemicals used for the dewatering operation. Sulfates were not detected in the digestates, whereas sulfides were only present in MK2 with an average concentration of 1550.00 μg/L. The concentration of chloride was at a relatively low and at a comparable level in all of the materials. Likewise, the amounts of metals were at similar levels except for aluminum, which was found at much greater concentrations in the MK1 and MK2 digestates. Out of the three tested liquid digestates, the MK2 substrate was selected for further investigations mainly due to having the greatest amount of nitrogen and having a high pH.

### 2.2. Isolation and Taxonomic Identification of Microalgae

**Figure 1:**
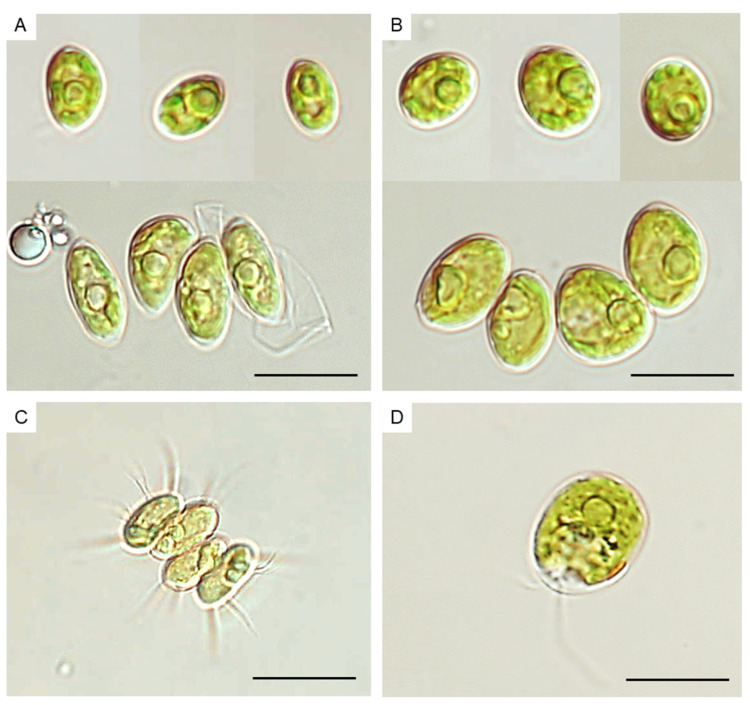
Microalgae from the mixed algal culture. (**A**) *Tetradesmus obliquus* PNKW01; (**B**) *Tetradesmus obliquus* PNKW04; (**C**) *Desmodesmus subspicatus* PNKW02; (**D**) *Microglena* sp. PNKW03. Scale bars: (**A**–**C**) 20 µm; (**D**) 15 µm.

Using the MAU-D (Microscope Assisted Unialgal isolation through Dilution) method for the isolation of microalgae from the environment according to Section 3.2.1, no unialgal non-axenic cultures were obtained; therefore, we only observed mixed microalgae cultures. However, the microorganisms present in the cultures were initially classified as *Tetradesmus* sp., *Desmodesmus* sp., and *Chloromonas* sp., and in the isolated unialgal cell lines, four morphotypes were observed. Two strains, PNKW01 and PNKW04, were morphologically similar (Figure 1A,B); however, the first had less obtuse cells. In unialgal cultures, both strains occurred in the dominant single-cell form and sometimes in the four-celled coenobium. The strain PNKW02 (Figure 1C) was mostly composed of four–celled coenobium with long bristles arising from both the apical parts of cells in the coenobium and lateral parts of the marginal cells. The PNKW03 strain was composed of singular motile cells with two visible flagella (Figure 1D). The morphological features and molecular hits from BLASTN analysis indicated *Tetradesmus obliquus* species in the PNKW01 and PNKW04 strains and *Desmodesmus* sp. in the PNKW02 strain. For PNKW03, both the BLASTN results and traditional identification was insufficient for species identification (=*Chloromonas*/*Carteria*/*Microglena*).

**Figure 2:**
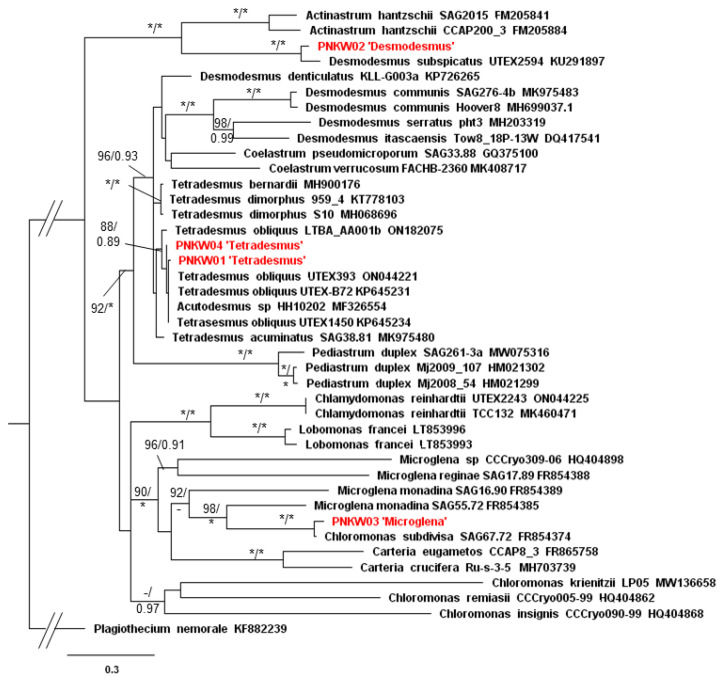
Phylogenetic tree of microalgae with *Plagiothecium* (bryophyte) as the outgroup taxa based on the nuclear ITS markers (1363 bp total). The tree shows the position of the studied strains among the green microalgae. Numbers on the branches indicate bootstrap values from ML followed by posterior probabilities from the BI analysis. Asterisk (*) indicates 100 (ML) and 1.00 (BI) values, while negative values (−) indicate values below 90 (ML) and 0.90 (BI). Names of taxa were verified according to the recent taxonomic changes with the use of the AlgaeBase database [17]. The topology of the tree was based on the ML analysis.

**Figure 3:**
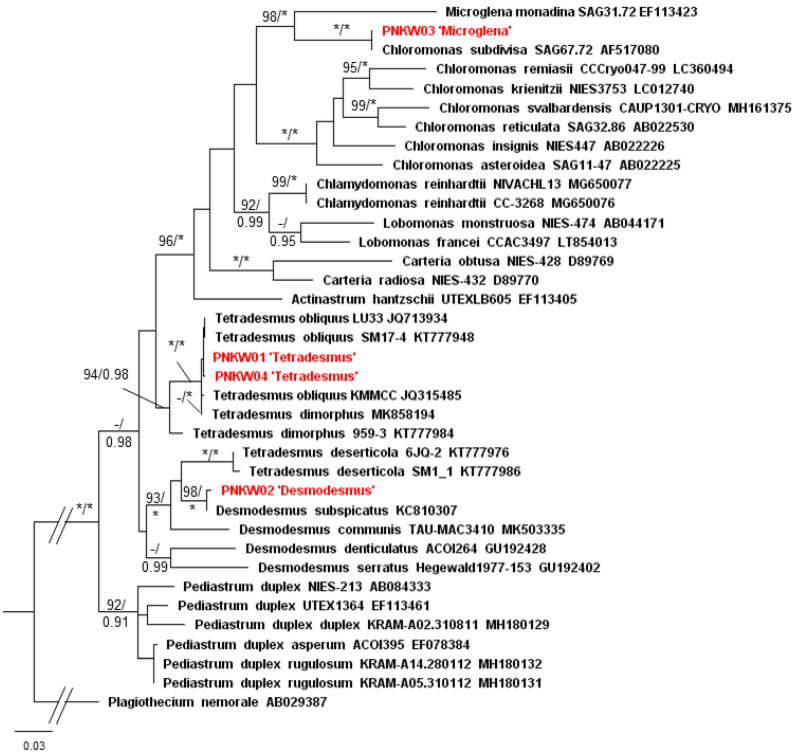
Phylogenetic tree of microalgae with *Plagiothecium* (bryophyte) as the outgroup taxa based on the *rbcL* marker (total 698 bp). The tree shows the position of the studied strains among the green microalgae. Numbers on the branches indicate bootstrap values from ML followed by posterior probabilities from BI analysis. Asterisk (*) indicates 100 (ML) and 1.00 (BI) values, while negative values (−) indicate values below 90 (ML) and 0.90 (BI). Names of taxa were verified according to the recent taxonomic changes with the use of the AlgaeBase database [17]. The topology of the tree was based on the ML analysis.

Molecular analyses of PNKW01 and PNKW04 confirmed the identification of the species (Figure 2 and Figure 3). Both the ITS and *rbcL* matrix strains were placed in the clade of *Tetradesmus obliquus*; however, the maximum branch support was obtained in the case of the cp marker (BS = 100 and PP = 1) (Figure 3). For PNKW02, a *Desmodesmus* species, the ITS and *rbcL* gene were closely related to the *D. subspicatus* strain UTEX2594 with respect to the ITS region and *D. subspicatus* LUCC011 with respect to the rbcL. The morphology of *Desmodesmus* cells together with molecular data confirm that in the mixed culture species, *D. subspicatus* occurs. In the case of PNKW04, both the ITS and *rbcL* phylogenetic analyses placed the strain next to *Chloromonas subdivisa* SAG67.72 with a maximum branch support. However, both taxa were a part of a well-supported clade of the *Microglena* genus, which is phylogenetically distinct to *Chloromonas* clade (Figure 2 and Figure 3). Taking into consideration the morphological features of PNKW03 and its phylogenetic position, it is highly probable that the strain from the mixed culture is a representative of the *Microglena* genus.

### 2.3. Calibration of Maximum Absorbance for Optical Density

The wavelength at which the optical density measurement is performed depends on the tested microorganisms. When examining the growth of microalgae by spectrophotometry, it is recommended to measure the absorbance in the range of 664–690 nm, which is correlated with the wavelength of light captured by chlorophyll [18,19]. A wavelength scan was carried out for the 11 microalgae breeding cultures reported in Section 3.2.1. Additionally, we conducted scans for two microalgae cultures cultivated in bioreactors at the beginning stage of experiment, as according to Section 3.2.3. (after the multiplication stage, without the addition of liquid digestate). The scan was performed within the absorption spectrum of microalgae in the range of 540–900 nm and gave the maximum absorbance of 680 nm for all samples. The obtained value is similar to the optimal absorbances reported in the literature [19,20].

### 2.4. The Growth Curves for Isolated Microalgae

**Figure 4:**
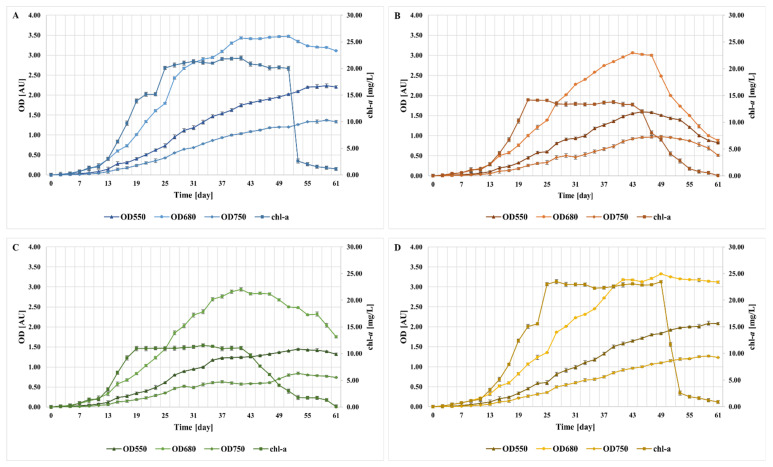
Growth curves of the mixed microalgae cultures (**A**–**D**). Each plot corresponds to the particular cultures designated as (**A**–**D**).

Based on the concentration of chlorophyll *a* (chl-*a*) and optical density (OD), the growth curves for randomly selected microalgae cultures A–D were plotted (Figure 4). In all of the investigated microalgae cultures, four growth phases were observed: lag phase, log phase, stationary phase, and death phase. In each of the microalgae cultures tested, the concentration of chl-*a* was close to zero for the first 3 days of the process. This initial stagnation phase is typical for microalgae. During this phase, cells adapt to the culture conditions, increasing the level of metabolites and enzymes needed for C fixation and cell division [21,22]. Depending on the culture, the exponential growth phase lasted from 16 to 22 days. The two cultures marked as (A) and (D), respectively, had the longest time of cell division (up to 25 days of breeding) in which an increase in population density was observed. As soon as light, nutrients, and other physical-chemical factors began to limit the multiplication of microalgae, the two cultures entered a phase of stationary growth, during which cell density was relatively constant [22,23]. For culture (A), a stationary phase was observed from day 25 to day 51 of the process, after which the culture entered a death phase of >10 days. On the same day, the stationary phase started in culture (D), and was 2 days shorter compared with culture (A). The shortest stationary phase of 22 days was observed for culture (B) and (C), followed by a death phase of 18–20 days. The correlation between the concentration of chl-*a* and the optical density at 680 nm was only recorded at the moment of reaching the death phase by the investigated microalgae cultures. The decrease in both OD680 (optical density at 680 nm) and chl-*a* occurred on the same day of the run. In contrast, the OD values at 550 and 750 nm practically increased for the entire cultivation processes of (A), (C), and (D), with only a slight drop on day 59. For culture (B), the decrease in OD550 and OD750 was observed after day 45 and day 49 of the process, respectively (Figure 4).

In the stationary phase, culture (A) and (D) were characterized by a chlorophyll *a* concentration in the range of 20.00–21.93 mg/L and 22.25–23.49 mg/L, respectively, thus achieving higher concentrations of green pigment compared with culture (B) and (C). Additionally, they were characterized by a longer stationary phase of 24–26 days (Figure 4). Due to the high chlorophyll *a* content and long stationary and log phases, culture (A) and (D) were selected for the treatment of the MK2 liquid digestate in the further step of experiment. Culture (A) and (D) were multiplied in B1 and B2 photobioreactors, respectively.

### 2.5. Treatment of the Liquid Digestate in Photobioreactors

**Figure 5:**
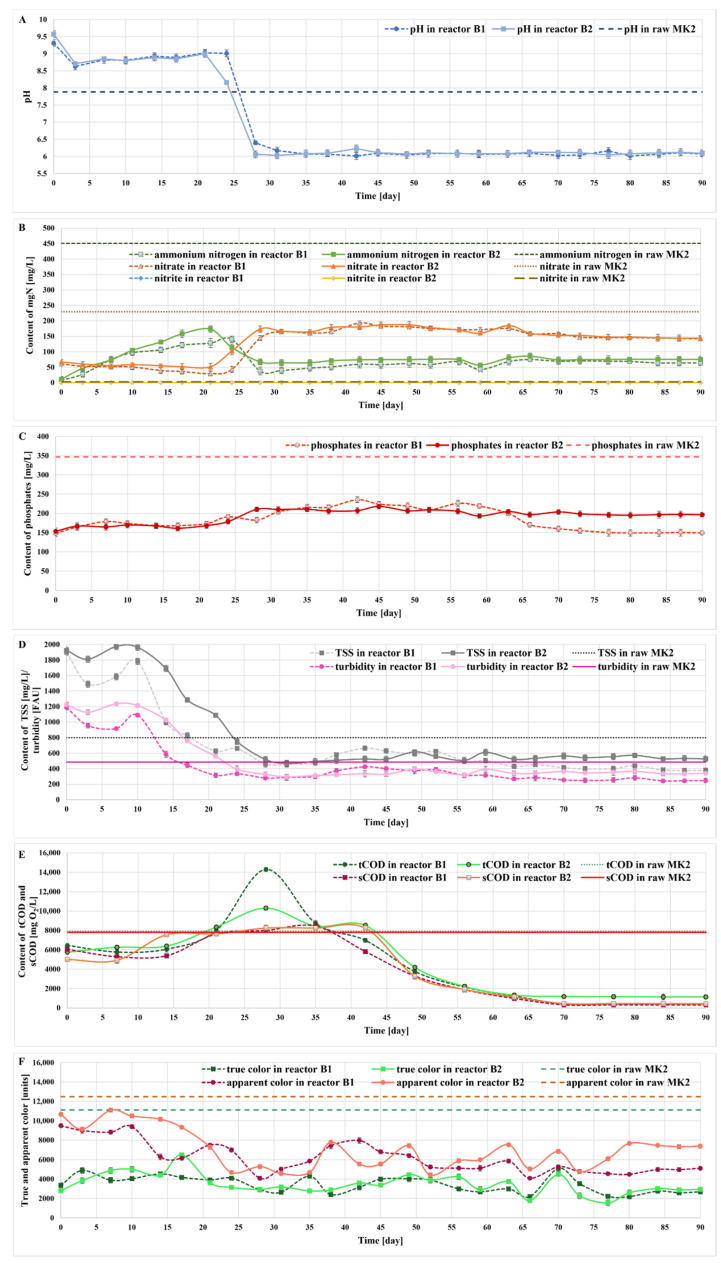
Changes in the pH (**A**), ammonium nitrogen, nitrates, nitrites (**B**), phosphates (**C**), total COD, TSS, turbidity (**D**), soluble COD (**E**), and color (**F**) during the 90-day digestate treatment process in the photobioreactors.

The efficiency of removing organic contaminants and nutrients from the MK2 liquid digestate was investigated in two photobioreactors using consortia of the microalgae *Tetradesmus obliquus*, *Desmodesmus subspicatus*, and *Microglena* sp. according to the methodology described in Section 3.2.3. After a 30-day incubation period with a 3N-BBM medium, the reactors were semi-continuously fed MK2 digestate for 90 days. The data of these experiments are illustrated in Figure 5.

Nitrogen is one of the most important nutrients for the growth of microalgae. Algae have been documented to be capable of using various nitrogen sources, including ammonium nitrogen, nitrite, nitrate, or urea [24]; for these microorganisms, nitrates are a preferred form of nitrogen form to begin with. This can be explained by the fact that nitrates are the most energy-efficient nitrogen source, requiring a small amount of energy for its uptake [25]. In this study, three forms of nitrogen were monitored throughout the semi-continuous runs, namely N-NH_4_, N-NO_3_, and N-NO_2_ (N). Ammonium nitrogen was the main nitrogen form in the raw liquid digestate, accounting for 66% of the total nitrogen. Ammonia is recognized as a buffering agent that increases the pH of the liquid environment. For the first 3–4 weeks of experiments with the liquid digestate, the changes in both ammonium nitrogen and pH were observed in both photobioreactors during which the microalgae adapted to new, difficult conditions. After 24 and 21 days of cultivation, respectively, there was a reduction in the pH and ammonium nitrogen content, while the concentration of nitrates sharply increased, which is likely associated with the growth of nitrifying bacteria and general remodeling of the microflora (biological succession process). The concentration of nitrite throughout the experiments was kept at a constant, low level. After the period of adaptation, the process conditions in the photobioreactors stabilized. In the experiments that we conducted, a complete removal of nitrogen from the digestate was not achieved. The removal rates were calculated based on the results from the last 5 weeks of the experiments, during which the reactors maintained a steady-state operation. Hence, the reduction rate of nitrogen reached 70% in comparison with the raw MK2 digestate (Figure 5B). A similar efficiency was also reported by Xu et al. [13], who used autoclaved and diluted piggery wastewater as a culture medium for *Tetradesmus obliquus*. Using the same species of microalgae for the treatment process, Massa et al. [15] achieved a N-NH_4_ reduction rate of up to 99.8%; however, the liquid digestate was diluted and autoclaved to reduce its turbidity and to eliminate other organisms before being used for experiments. A dilution of the digestate was also carried out by Franchino et al. [26]. Depending on the dilution degree, *Tetradesmus obliquus* lowered the N-NH_4_ level by 83.7–92.4%. Moreover, Ji et al. [27] investigated *Desmodesmus* sp. for the treatment of diluted wastewater. In batch experiments, the reduction rates of the total nitrogen and N-NH_4_ were 75.6% and 92.7%, respectively, whereas the corresponding values achieved in fed-batch experiments were 94.2% and 91.1%, respectively.

Phosphorus, similar to nitrogen, is an essential nutrient for the growth of microalgae. Phosphates constitute up to 82–90% of the total phosphorus (TP) in digestate and are necessary for many different microalgal cellular processes, such as the synthesis of nucleic acids and energy transfer [4]. Phosphates can be removed from the digestate by microorganisms through various mechanisms. Phosphate ions can be fixed to biomass [28], adsorbed on the cell surface [29], or removed via chemical precipitation [30]. Phosphates at the average concentration level of 346.67 mg/L were recorded in the MK2 effluent. For a long period of the experiment, the level of the indicator remained at a constant level. However, it could be noticed that with the decrease in pH and ammonium nitrogen, both nitrates and phosphates increased. A marked drop in phosphates was noticed after a few weeks of the treatment process. The removal rate of phosphate in the steady-state period reached 43–57% (Figure 5C). Massa et al. [15], using *Tetradesmus obliquus* for the treatment of liquid digestate, achieved a P-PO_4_ reduction rate of 86.1%. A similar treatment efficiency (71–89%) was also reported by Xu et al. [13] for autoclaved and diluted piggery wastewater using the same microalgae. Moreover, Ji et al. [27] used diluted wastewater as a source of nutrients for *Desmodesmus* sp. and observed a reduction of P-PO_4_ ranging from 88.7% in fed-batch experiments to 100% in batch experiments. Furthermore, Franchino et al. [26] applied an agrozootechnical digestate as the growth medium and reported a 94.4% P-PO_4_ removal rate by *T. obliquus*.

Most of the studies focus on the removal of nutrients from the digestate, whereas the removal of organic carbon (expressed by COD) by microalgae is rarely described. In this study, after a 30-day initial experimental period, the total COD (tCOD) values measured in photobioreactor B1 and B2 were 6450 mg O_2_/L and 5750 mg O_2_/L, respectively, whereas the corresponding values of soluble COD (sCOD) were 6040 mg O_2_/L and 5010 mg O_2_/L, respectively. During the following 14 days, the tCOD remained at a constant level and then increased up to 14,300 mg O_2_/L (B1) and 10,300 mg O_2_/L (B2) on day 28. Then, a gradual decrease in the COD was observed, which corresponded with a stabilization of most indicators measured in the reactors. In the final experimental period (last 5 weeks), a reduction in the total and soluble COD in reactor B1 reached 95% and 96%, respectively, whereas the corresponding values for B2 were 85% and 94% in comparison with the raw MK2 digestate (Figure 5E). These figures are higher than the values reported in the literature. For instance, Xu et al. [13] achieved up to 75% of COD reduction using autoclaved and diluted piggery wastewater as a medium for *T. obliquus*. Prajapati et al. [31], using cyanobacterium *Chroococcus* sp. for the treatment of diluted liquid digestate, reported an sCOD reduction rate of approximately 70%.

Considering turbidity and total suspended solids (TSS), their concentrations were the highest in the initial acclimation period. Then, both indicators gradually decreased and achieved constant values on day 28. In the final experimental period (last 5 weeks), the values of turbidity were reduced by 48% (reactor B1) and 29% (reactor B2), whereas the TSS was reduced by 50% in B1 and 32% in B2 compared with the raw MK2 digestate (Figure 5D). However, it must be noted that both the turbidity and TSS are affected by the liquid digestate on the one hand, and by the algal biomass on the other. Microalgae form flocculent suspensions but also remove particles suspended in the liquid digestate, thus partially reducing both the TSS and turbidity [4]. Similarly, color is also affected by both the liquid digestate and algal biomass growing in the photobioreactors. The average values of true color and apparent color in the raw MK2 liquid digestate were 11,117 units and 12,483 units, respectively. The biological treatment allowed for a significant reduction in especially true color by 76% (B1) and 77% (B2) in comparison with the raw MK2 digestate. There was a reduction rate in the apparent color at 61% in B1 and 46% in B2 compared with the raw MK2 digestate. Interestingly, both the true and apparent colors remained at relatively constant levels throughout the experiments (Figure 5F).

### 2.6. Monitoring the Growth of Microalgae in Bioreactors

**Figure 6:**
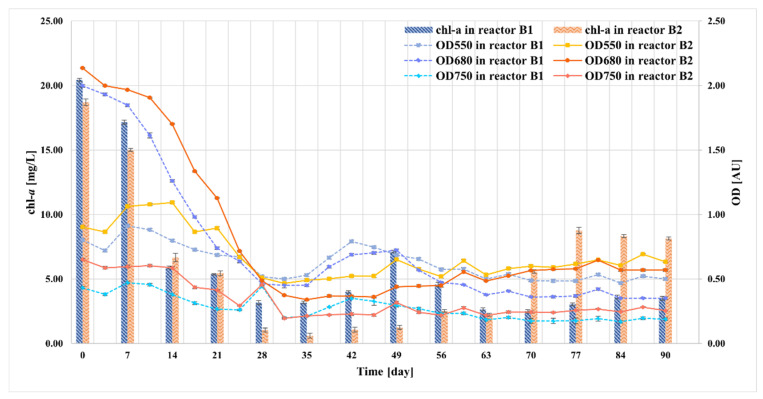
Change in the chlorophyll *a* concentration and optical density at 550, 680, and 750 nm during the treatment of the liquid digestate.

The growth of microalgae in the bioreactors was monitored by two indicators: the concentration of chl-*a* and optical density at 550 nm (OD550), 680 nm (OD680), and 750 nm (OD750) (Figure 6). After a month of microalgae multiplication in 300 mL of 3N-BBM medium, the concentration of chlorophyll *a* was measured at 20.44 mg/L in the B1 bioreactor and 18.70 mg/L in the B2 bioreactor. The optical density at a wavelength of 680 nm was correlated with the concentration of chlorophyll *a*. OD680 decreased with the decrease in chl-*a* concentration, and vice versa. After 14 days of the experiments, a sharp reduction in chl-*a* concentration was observed in the order of 71% (B1) and 64% (B2) with respect to its original value. This also correlated with a sharp decrease in the OD680 indexes of both bioreactors. After 35 days of experimentation, the values of optical density stabilized with only slight changes, whereas chl-*a* showed a more visible variations. In addition, the concentration of chlorophyll *a* and the microalgal cell density showed that microalgal biomass productivity reached a constant level at the 63rd and 77th day of the process in photobioreactor B1 and B2, respectively. The optical density at 550 nm, corresponding to the minimum absorption of chlorophyll and minimizing changes in the chlorophyll concentration, was also measured during the semi-continuous runs. The OD550 values in both photobioreactors varied in the first experimental period, with two peaks observed on day 14 and 42. This indicator remained stable from day 63 on. In addition, the optical density was measured at 750 nm, and this wavelength is regarded as the most independent regarding changes in the content of photosynthetic pigments in microalgal biomass. The changes of OD750 were comparable to the ones observed for OD550, suggesting that both indicators measured the presence of impurities (Figure 6).

### 2.7. Qualitative and Quantitative Structure of Microalgal Culture in Bioreactors

**Figure 7:**
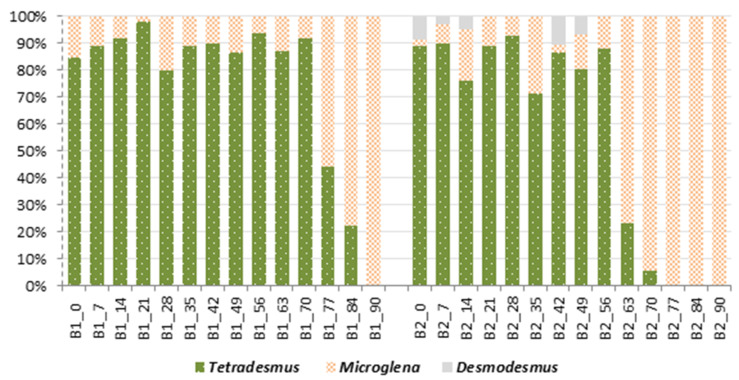
Percentage share of taxa in bioreactors during biological treatment; bioreactor B1 and B2 with the subsequent days of treatment.

From the beginning of the experiment, the cultures in both bioreactors were dominated by *Tetradesmus obliquus* cells (Figure 7). Both cell morphotypes of the species—more and less obtuse—were present, and the species occurred in single-celled and coenobium form types. In the last month of treatment, the structure of the consortium shifted; *Microglena* sp. cells dominated the biomass of both reactors. This phenomenon began earlier in bioreactor B2—between day 56 and 63 of the experiment; in B1, the phenomenon occurred between day 70 and 77. The presence of *Desmodesmus subspicatus* was only noted in the B2 bioreactor, and this species randomly co-occurred in consortium in the first and second month of the experiment.

*Tetradesmus obliquus* growth highly depends on a proper environmental pH. The most optimal pH is around 7 [32], and deviations above or below 7 decrease the rate of cell division [33]. Here, a rapid drop in the pH of eluent by ca. 3 was noticed between day 21 and 28 of the experiment in both bioreactors, resulting in a decrease in chl-*a* concentration by 41% in B1 and 81% in B2. As El-Sheekh et al. [34] noticed, the growth of *Tetradesmus* sp. in a pH of 6.5 is lower than in the optimal 7.0 by ca. 30%; however, the cells try to reproduce, and after 18 days, the optical density of the culture begins to decrease. A lower pH, such as 5.5, almost stops the growth of this microalga [34]. Unfortunately the preferences of *Microglena* sp. in PNKW03 with regard to pH has not yet been established. After day 28, the pH in both bioreactors was not favorable for the *Tetradesmus obliquus* culture and some fluctuations between *Tetradesmus* sp. and *Microglena* sp. were noticed until day 70 in B1 and day 56 in B2, without any important trend. To understand the change in the bioreactors’ compositions at the end of experiment, it is essential to keep in mind that factors affecting the growth of microalgae are very complex and act together, even in a small-scale culture. It is well-known that both the pH value and nutrient concentration, often expressed as the N-NO_3_ to P-PO_4_ (N:P) ratio, are extremely important for the growth of phototrophs. Considering the B1 reactor, the chl-*a* concentration was lower than in the B2 reactor, and in day 70–73, a small decrease in the N:P ratio was observed. This, together with an unfavorable pH for the *Tetradesmus* sp., could be the impulse for shifting the proportion between both species towards the *Microglena* sp. Moreover, such a potential explanation works better in the case of B2, where from day 49 on, the N:P ratio gradually decreased with a small increase between day 56 and 59 (specifically as an increase in the percentage of *T. obliquus*). In both bioreactors, there is evidence that the dominance of *Microglena* could be the result of too low of a pH and N/P ratio for *Tetradesmus* growths.

## 3. Materials and Methods

### 3.1. Environmental Sampling and Media

The samples of water from the artificial eutrophic pond (Stawy Jana, Lodz, Poland; 51°42′37.774″ N, 19°28′58.254″ E) were collected in 2021 and stored in glass bottles exposed to sunlight at 20–25 °C as the start-up culture.

Digestates named MK1 and MK2 were derived from the anaerobic digestion pilot installation for treating vegetable wastes at Warmia Fruit and Vegetable Processing Company Ltd. in Kwidzyn, Poland. The installation consists of two reactors, each with a working volume of 50 L that treat the mixture of corn waste (grains, leaves, stalks, and cobs) with green peas. The digestate MK1 was collected from reactor 1, which operated at an organic loading rate (OLR) of 1 kgVS/m^3^/d; the digestate MK2 was derived from reactor 2, which operated at an OLR of 1.5 kgVS/m^3^/d. Both digestates were then centrifuged at 4000 rpm (4800 G) using a Rotina 420 centrifuge (Hettich, Bäch, Switzerland) to obtain liquid fractions for the experiments. The third liquid digestate named GS was obtained from the anaerobic digester (after a dewatering of the digested sludge) from the municipal wastewater treatment plant in Lodz, Poland. The characteristics of the liquid digestates are summarized in Table 1.

For the purpose of microalgal cell isolation and cultivation, a 3N-BBM medium was used. The medium was prepared according to the methodology described in Andersen [33]. The final pH was set to 6.6 ± 0.2.

### 3.2. Experiments

#### 3.2.1. Isolation of Microalgae from the Environment

The Microscope Assisted Unialgal isolation through Dilution (MAU-D) method was used to isolate the microalgae in the first stage of the experiment [35]. In brief, 20 µL of start-up culture containing green algae, cyanobacteria, and diatoms was placed on a sterile glass slide next to 6–8 drops of sterile 3N-BBM medium in a volume of 15 µL. Then, 5 µL of the start-up culture was introduced into the first drop of 3N-BBM and, via passaging through a series of dilutions, the final drop containing few cells of the same morphotype was pipetted from the slide to 100 mL of sterile 3N-BBM medium. The isolation was monitored at a magnification of 100× using a light microscope (Delta Optical, Warsaw, Poland). New cultures (breeding cultures, n = 11) were incubated for 24 h without stirring in a dark room, then for 21–30 days at 25 °C with magnetic stirring (Multistirrer 6, Alchem, Lodz, Poland) and then illuminated with two LED lamps (T8 LED Linio model), each having 14.5 W of electric power, 1440 lm of luminous power, and a color temperature of 6000 K (cold white color). The intensity of illumination was set at 2200 Lux, which was measured with a luxmeter LXP-10B (Sonel, Swidnica, Poland). A photoperiod of 14/10 h (light/dark hours) was applied to all experiments. Subsequently, microscopic observations of microalgal cultures were performed using a light microscope (Delta Optical, Warsaw, Poland), and the microalgae were initially identified.

#### 3.2.2. Determination of the Growth Curves for Isolated Microalgae

The growth curves were determined for four randomly selected breeding cultures, designated as culture A–D. The growth of the cultures was monitored using two variables: the chlorophyll *a* concentration and optical density measurements. Data were collected every 48 h for 61 days under the same conditions of cultivation as described in Section 3.2.1 until the cultures reached the dieback phase.

#### 3.2.3. Liquid Digestate Treatment by Mixed Microalgae in Bioreactors

The treatment efficiency of the liquid digestate by the microalgae cultures was established using a specially designed installation, which consisted of two bioreactors, each with a capacity of 350 mL, which were operated semi-continuously. The reactors were illuminated with two LED lights (T8 LED Linio model) with the parameters described in Section 3.2.1, which were mounted at a distance that allowed for the light intensity at 2200 Lux to be maintained. The photoperiod was set at 14/10 (light/dark hours) throughout the experiments. The bioreactors were aerated at a flow rate of 0.7 L/L/min using a Thomas AP-80H membrane pump model (Yasunaga, Tokyo, Japan). The start-up of the reactors was performed with two microalgae cultures, selected from the A–D flasks with breeding cultures, according to the results of the growth measurements (best physiological activity of cells, long stationary, and log phases of culture). The cultivation in bioreactors was initially set for 30 days without feeding, and then the reactors were semi-continuously fed with the digestate that had the best properties for the experiment (MK2—see Section 2.1). The hydraulic retention time (HRT) was set at 30 days. During the digestate treatment, the physical and chemical analysis of the liquid were performed along with the algal biomass monitoring. Most of the analyses were performed twice a week, except for the COD, concentration of chlorophyll *a*, and abundance of cells, which were measured once a week.

### 3.3. Analyses

#### 3.3.1. Physical and Chemical Analyses of Liquid Digestate

The total solids (TS), volatile solids (VS), and pH were analyzed according to the *Standard Methods for Testing Water and Wastewater* [36]. The total volatile fatty acids (TVFA) and total and soluble chemical oxygen demand (tCOD and sCOD) were determined using a UV-VIS DR6000 spectrophotometer (Hach Lange, Loveland, CO, USA) and Hach Lange test LCK365 and LCK 214, respectively. The following Hach Lange tests were also used: a modified Nessler method (no. 8038) for ammonium nitrogen (N-NH_4_), the NitraVer 5 and NitriVer 2 tests for nitrates (N-NO_3_) and nitrites (N-NO_2_), a PhosVer 3 method (no. 8048) for phosphates (P-PO_4_), a SulfaVer 4 method (no. 8051) for sulfates, a methylene blue method (no. 8131) for sulfides, and a mercuric thiocyanate method (no. 8113) for chlorides. The determination of metals was performed with the following Hach Lange tests: a CuVer 1 method (no. 8506) for copper, a FerroVer method (no. 8008) for iron, a Zincon method (no. 8009) for zinc, and an AluVer method (no. 8012) for aluminum. The true color, apparent color, total suspended solids (TSS), and turbidity were assessed by measuring the absorbance at 455 nm, 455 nm, 810 nm, and 860 nm using a UV-VIS DR6000 spectrophotometer. All analyses were performed in triplicates, and the results were presented as the mean values with the standard deviation.

#### 3.3.2. Calibration of Maximum Absorbance for Optical Density Measurement

Because the optical density mostly results from light scattering, the size and shape of cells are key modifiers affecting the results; we performed the calibration of the maximum absorbance for cultures in the experiment using these parameters. In order to analyze the growth rate of the microalgae, the maximum absorbance for each sample was determined by scanning the culture in the stationary phase within the range of 540–900 nm using a UV-VIS DR6000 spectrophotometer. The maximum value of absorbance was then selected to estimate the growth curves by optical density [37].

#### 3.3.3. Optical Density Measurement

The effectiveness of the biomass growth of microalgae cultures was assessed by measuring the OD at three wavelengths, 550 nm, 680 nm, and 750 nm. The analyses were performed using a UV-VIS DR6000 spectrophotometer. Samples were taken immediately before the measurement and placed in cuvettes with an optical path length of 10 mm. The mixed MK2 digestate was used as a blank. The wavelength, which correlated with chlorophyll absorbance, was set at 680 nm, determined experimentally as described above (see Section 3.3.2). Additionally, the optical density was measured at a wavelength of 550 nm, which corresponded to the minimum absorption of chlorophyll, and this indicator minimizes the effects of changes in green pigment concentration [38]. The absorbance measurement was also taken at 750 nm. This wavelength is not specific for microalgae, but its use avoids light absorption by chlorophyll and carotenoids and remains independent of the changes in the photosynthetic pigment content that occur in the microalgae biomass [18].

#### 3.3.4. Measurement of the Chlorophyll *a* Concentration

The concentration of chlorophyll *a* (chl-*a*) was measured following the method described by [39]. Each microalgae culture was introduced into two Eppendorf tubes with a volume of 1.3 mL and centrifuged (1 min at 6000 rpm) using a centrifuge MPW 380 R (MPW, Cracow, Poland). The supernatant was removed, and the cells were resuspended in 100 µL of 90% methanol. The samples were then homogenized with a handheld homogenizer (EURx, Gdansk, Poland) for 30 s. The samples were incubated in the dark for 24 h at 4 °C for pigment extraction; then, the cell debris was separated by centrifugation (1 min at 6000 rpm). The absorbance of the sample was measured at 650 nm and 665 nm using 90% methanol as a blank. The concentration of chl-*a* was calculated according to Equation (1):

Chl-*a* (mg/L) = (16.5 × A_665_) − (8.3 × A_650_) (1)

#### 3.3.5. Taxonomic Analyses of Microalgae from Cultures

After the initial isolation of microalgae using the MAU-D method, which allowed us to obtain only mixed cultures of microalgae and adapt them to the process of liquid digestate purification, we carried out an isolation of cells by micromanipulation to obtain pure unialgal cell lines for molecular identification purposes. From a mixed cell culture, a single cell of particular morphotypes was isolated by passaging using a microcapillary into unialgal non-axenic cultures. Cells were transferred via a sterile 3N-BBM medium using a mouth aspirator and microcapillary while being observed under a NIKON Eclipse TS100 inverted microscope (Precoptic Co., Warsaw, Poland). Pure cultures were incubated under the conditions that were previously described (see Section 3.2.1).

Four microalgae strains, designated as PNKW01–PNKW04, were obtained through the isolation procedure and were deposited in the collection of the Department of Algology and Mycology, University of Lodz. The taxonomic analysis was performed using both traditional and molecular approaches. The initial identification was based on the cell’s morphology and morphometric features under a Nikon Eclipse 50i light microscope with DIC optics (Precoptic Co., Warsaw, Poland) using the taxonomic reference of Ettl and Gärtner [40]. Microphotographs of the strains were taken using an OPTA-Tech digital documentation system. The molecular approach involved the phylogenetic analyses of cultured strains and closely related microalgae based on the nuclear ‘ITS’ marker (from the 3′ end of the nuclear spacer ITS1, through the 5.8S rDNA, to the 5′ end of the ITS2 spacer); and chloroplast *rbcL* marker encoding the Rubisco large subunit.

The total DNA was extracted using the DNeasy Plant Mini Kit (Qiagen, Germantown MD, USA), where we followed the manufacturer’s protocol of three equal samples for each strain. DNA extracts were quantified with a BioDrop DUO Spectrophotometer (BioDrop Ltd., Cambridge, UK), and the samples with high-quality DNA (1.7–1.9 OD260/OD280) were selected for further analyses. All markers were amplified by the PCR for a nuclear marker in two overlapping fragments, while the cp marker was amplified as a single amplicon. The PCR reactions were conducted using the OptiTaq PCR Master Mix (Eurx, Gdansk, Poland) and dedicated primers (Table 2). Amplicons were cleaned using the Syngen Gel/PCR Mini Kit (Syngen Biotech, Wroclaw, Poland) according to the manufacturer’s protocol and were sequenced using Sanger sequencing and primers from the amplification process of SEQme s.r.o. company (Dobris, Czech Republic). The obtained sequences were then assembled in the Geneious 11.1.5 software (Biomatters Ltd., Auckland, New Zealand) and submitted to the NCBI GenBank database (www.ncbi.nlm.nih.gov, accessed on 20 April 2022) under the accession number ON426490-ON42693 for ITS and ON457158-ON457161 for *rbcL*. The remaining sequences were compared with the available sequences in NCBI using the BLASTN analysis [41] (http://www.ncbi.nlm.nih.gov/, accessed on 20 April 2022). Sequences were aligned using the MAFFT v.7 web server [42] (http://mafft.cbrc.jp/alignment/server/, accessed on 23 April 2022), where the auto strategy was applied, with the settings of a scoring matrix of 200PAM, gap opening penalty of 1.53, UniREf50 for Maft-homologs, and plot and alignment with a threshold of 39 score. The obtained alignments were checked for poorly and ambiguously aligned regions and small corrections were made from visual observations. The evolutionary models were calculated using the PartitionFinder 2 software [43] and were chosen according to the Akaike Information Criterion (Table 3).

Phylogenetic calculations were performed using the maximum likelihood analysis (ML) in the IQ-TREE web server [49] (http://iqtree.cibiv.univie.ac.at/, accessed on 25 April 2022) using the ultrafast bootstrap (UFBoot) pseudolikelihood algorithm [50] and 10,000 replicates; we also utilized Bayesian inference (BI) in MrBayes 3.2.2 [51], where two parallel Markov chain Monte Carlo (MCMC) runs for four million generations each, with trees sampled every 1000 generations. The average standard deviation of the split frequencies in both cases remained below 0.01 for the last 1000 generations, and posterior probabilities were estimated from the 50% majority-rule consensus tree after the elimination of the first 25% of samples as a burn-in. The alignment and tree files were submitted to the Figshare online database (https://doi.org/10.6084/m9.figshare.20176151, accessed on 1 September 2022).

#### 3.3.6. The Structure of Microalgae Culture in Bioreactors

The qualitative and quantitative structure of microalgae in bioreactors during liquid digestate treatment were monitored. The percentage share of each taxa in total biomass was assessed based on the abundance of cells per 1 mL of culture. The abundance was calculated using the droplet method on microscope slides and following Equation (2):

Lx = (ni × Pp)/(Pi × V)(2)

where Lx is the abundance of cells per 1 mL of culture, ni is the number of cells of each taxa counted, Pp is the surface area of the coverslip (400 mm^2^), Pi is the surface area viewed for counting under the microscope (mm^2^), and V is the volume of the sample applied to a microscope slide (cm^2^).

For the purposes of the analysis, only the cells that appeared microscopically healthy and physiologically active were counted; those that had a damaged photosynthetic apparatus or were partially decomposed were omitted from the calculation.

## 4. Conclusions

This study demonstrated that liquid digestates after an anaerobic digestion of plant organic waste can successfully be treated by algae isolated from natural environments. The consortium containing *Tetradesmus obliquus*, *Desmodesmus subspicatus*, and *Microglena* sp. could reduce the concentrations of nitrogen, phosphates, and the value of tCOD in the digestate by up to 70%, 57%, and 95% respectively. The use of environmental isolates rather than commercial strains for the treatment of digestates could be beneficial due to the potentially lower costs and increased tolerance of these organisms to high concentrations of pollutants.

## Figures and Tables

**Table 1:** Physical and chemical characteristics of the liquid digestates.

Indicator	Type of Liquid Fraction of Digestate		
	MK1	MK2	GS
pH	7.55 ± 0.01	7.88 ± 0.25	4.43 ± 0.01
Total Solids, TS [g/kg]	2.09 ± 0.01	3.23 ± 0.17	2.84 ± 0.01
Volatile Solids, VS [g/kg]	0.54 ± 0.01	1.73 ± 0.19	1.35 ± 0.02
Volatile Solids, VS [% TS]	25.91 ± 0.38	53.38 ± 3.19	47.58 ±0.37
Total Suspended Solids, TSS [mg/L]	185.00 ± 2.00	798.33 ± 158.62	7.33 ± 0.58
Turbidity [FAU]	129.00 ± 3.00	485.00 ± 111.00	111.00 ± 3.00
Apparent Color [units]	1631.00 ± 17.00	12,483.00 ± 2586.00	410.00 ± 1.00
True Color [units]	563.00 ± 1.00	11,117.00 ± 2835.00	369.00 ± 10.00
Total Chemical Oxygen Demand, tCOD [mg O_2_/L]	7743.00 ± 168.00	7900.00 ± 754.00	7413.00 ± 90.00
Soluble Chemical Oxygen Demand, sCOD [mg O_2_/L]	6810.00 ± 287.00	7800.00 ± 540.00	6417.00 ± 57.00
Total Volatile Fatty Acids, TVFA [mg/L]	3076.00 ± 70.00	3110.00 ± 1128.00	3097.00 ± 86.00
Ammonium Nitrogen, N-NH_4_ [mg/L]	131.33 ± 1.53	580.00 ± 95.50	128.67 ± 1.53
Nitrates (V), N-NO_3_ [mg/L]	766.67 ± 57.74	1016.67 ± 116.90	1013.33 ± 57.74
Nitrates (III), N-NO_2_ [mg/L]	5.67 ± 0.58	9.33 ± 3.01	6.00 ± 1.00
Phosphates, P-PO_4_ [mg/L]	360.00 ± 69.28	346.67 ± 26.58	810.00 ± 40.00
Sulfates [mg/L]	0.00 ± 0.00	0.00 ± 0.00	0.00 ± 0.00
Sulfides [μg/L]	0.00 ± 0.00	1550.00 ± 615.63	0.00 ± 0.00
Chlorides [mg/L]	330.00 ± 10.00	216.67 ± 36.70	283.33 ± 11.55
Iron [mg/L]	21.00 ± 4.00	4.00 ± 2.28	9.33 ± 0.58
Copper [mg/L]	5.67 ± 0.58	5.67 ± 0.52	7.00 ± 1.00
Zinc [mg/L]	10.33 ± 1.53	15.17 ± 1.72	14.00 ± 1.00
Aluminum [mg/L]	9.83 ± 0.40	10.30 ± 8.81	0.60 ± 0.20

**Table 2:** Primers used for amplification and sequencing with PCR reaction conditions.

Marker	Primer	F/R	Reference	Reaction Conditions
ITS	ITS1AKL	F	[44]	95 °C (3 min) 35 × [94 °C (1 min)/52 °C (1 min)/72 °C (2 min)] 72 °C (7 min)
	Nr-LSU-0012-3′	R	[45]	
	ITS3	F	[46]	
	HLR3R	R	[47]	
*rbcL*	rbcL1	F	[48]	95 °C (2 min) 35 × [94 °C (1 min)/50 °C (1 min)/72 °C (3 min)] 72 °C (8 min)
	rbcL1421	R		

**Table 3:** Summary of partitions for the ITS matrix (1363 bp) and *rbcL* (698 bp); the evolutionary model selection and phylogenetic interference using PartitionFinder 2.

	ITS1	5.8S gDNA	ITS2	*rbcL* Codon
ML	TrN + I + G	TIM + I + G	JC	GTR + I
BI	F81	HKY	JC	GTR

## Data Availability

The sequences obtained during research were submitted to the NCBI GenBank database (http://www.ncbi.nlm.nih.gov/, accessed on 20 April 2022)under accession no. ON426490-ON42693 for ITS and ON457158-ON457161 for *rbcL*.

## Sample Availability

Not available.
